# Global and Chinese Burden of Inflammatory Bowel Disease From 1990 to 2021: A Systematic Analysis and Prediction of Disease Burden

**DOI:** 10.1002/jgh3.70160

**Published:** 2025-05-08

**Authors:** An Luo, Dongying Yao, Miao Wang, Liwen Jin, Zhihua Ran

**Affiliations:** ^1^ Department of Gastroenterology Zhou Pu Hospital Affiliated to Shanghai University of Medicine & Health Sciences Shanghai China

**Keywords:** China, epidemiological burden, global burden of disease (GBD), inflammatory bowel disease

## Abstract

**Background:**

Recent shifts in the global epidemiology of inflammatory bowel disease (IBD), particularly in emerging industrialized nations like China, underscore the need for in‐depth analysis.

**Methods:**

Utilizing the Global Burden Of Disease (GBD) 2021 database, we systematically examined IBD incidence, prevalence, death rates, and disability‐adjusted life years (DALYs) across Global, different social development index (SDI) regions, and countries from 1990 to 2021. Age‐standardized rates (ASR) and statistical metrics (APC, EAPC, AAPC) were employed to assess trends in IBD development, and a Bayesian age‐period‐cohort (BAPC) model was used to forecast future scenarios.

**Results:**

In 2021, the highest IBD incidence rates were observed in Canada (26.83/100000), Greenland (24.57/100000), and New Zealand (23.69/100000), markedly contrasting China's rate of 1.4/100000. Globally, IBD incidence increased modestly from 4.22/100000 in 1990 to 4.45/100000 in 2021 (EAPC = 0.29%). China experienced a more pronounced rise, with incidence jumping from 0.74/100000 to 1.4/100000 (EAPC = 2.93%). Notably, China also witnessed substantial declines in IBD deaths (56.00%) and DALYs (58.22%). The Middle SDI region exhibited a greater magnitude of change than Global and other SDI regions. The temporal trends in the incidence and prevalence of IBD in the countries are predominantly influenced by the period up to the year 2015. In China, between the ages of 15 and 49, the incidence and prevalence of IBD are projected to remain consistent with current standards, while the death rate and DALYs are predicted to exhibit a sustained decline.

**Conclusion:**

Despite the notable increase in IBD incidence in China, significant reductions in mortality and morbidity demonstrate the effectiveness of medical interventions and health system improvements.

## 1 Introduction

Inflammatory bowel disease (IBD) is a chronic, nonspecific, and progressive immune‐mediated inflammatory disease of the intestine, primarily including ulcerative colitis (UC) and Crohn's disease (CD). In recent years, the incidence of IBD has significantly increased worldwide, severely impacting patients' quality of life and social functioning [1]. According to the Global Burden Of Disease (GBD) 2019 data, the age‐standardized incidence rate in both sexes changed from 1.47 to 3.01 per 100 000 people in China from 1990 to 2019 [2]. This increase is closely related to China's rapid economic development and lifestyle changes. Westernized diets, diets high in fat and protein, increased life stress, and lack of exercise are considered significant factors contributing to the rise in IBD incidence [3]. Environmental pollution is also a crucial factor. During industrialization, environmental factors such as air, water, and soil pollution induce intestinal inflammation through various pathways, increasing the risk of IBD [4]. For example, the pollution levels in Beijing significantly correlate with the incidence of IBD, with each 10 μg/m^3^ increase in PM_2.5_ and PM_10_ concentrations associated with a 5% and 3% increase in IBD incidence, respectively [5].

Changes in gut microbiota are also essential factors in the onset of IBD. Modern lifestyles and the widespread use of antibiotics have altered the composition and function of gut microbiota, leading to gut dysbiosis and triggering IBD [6]. Significant progress has been made in gut microbiota research in high‐SDI (social development index) regions. Future research should focus on the characteristics of gut microbiota in emerging industrialized countries like China to find more effective prevention and treatment strategies. The improvement in healthcare also significantly affects the epidemiological characteristics of IBD. In China, with the advancement of medical technology and increased national investment in healthcare, more IBD patients can receive timely diagnosis and treatment. This has not only improved the survival quality of patients but also significantly reduced the death rate and DALYs(Disability adjusted life years) of IBD [7].

In summary, the epidemiology and burden of IBD are undergoing significant changes globally, particularly in emerging industrialized countries like China. Understanding these trends is crucial for developing effective public health policies and management strategies. This study aims to utilize the GBD 2021 database to analyze the burden of IBD in the world and China and predict the trends over the next decade to provide a scientific basis for the prevention and treatment of IBD.

## 2 Methodology

### 2.1 Data Source

The data for this study were sourced from the GBD 2021 database. The GBD database provides detailed health data for 204 countries and regions from 1990 to 2021, including Incidence rates, Prevalence rates, Deaths rates, and Disability‐adjusted life years (DALYs). We selected “Global”, “High SDI”, “High‐middle SDI”, “Middle SDI”, “Low‐middle SDI”, “Low SDI” and “China” as study areas, the SDI levels of different countries are shown in Table S1. We used “inflammatory bowel disease” as the study cause, selecting “Incidence”, “Prevalence”, “Deaths” and “DALYs” as study indicators. The measurement units included “number” and “rate” with gender categories of “female”, “male” and “both.” Age groups were classified as “age‐standardized” and “15–49 years” [8].

### 2.2 Data Processing

To ensure the comparability of the analysis results, we performed age‐standardized processing (ASR) on the data. ASR refers to the standardization of population data for specific age groups to eliminate the influence of age structure differences on the results. The formula for ASR is:ASR = (∑*iA*_*i*_ *w*_*i*_)/(∑_*i*_ *w*_*i*_) × 100 000 [9], where *A*_*i*_ is the age‐specific rate for the *i*‐th age group, and *w*_*i*_ is the population or weight corresponding to the *i*‐th age group in the standard population.

### 2.3 Indicator Interpretation

#### 2.3.1 Annual Percentage Change

Annual percentage change (APC) measures the trend of a health indicator over time. It is calculated based on a linear regression model, performing regression analysis on the natural logarithm‐transformed ASR. The formula is: APC = (e^β^ − 1) × 100 [10], where β is the slope of the natural logarithm‐transformed ASR in the regression model.

#### 2.3.2 Estimated Annual Percentage Change

Estimated annual percentage change (EAPC) is an advanced statistic based on APC, used to evaluate the trend of health indicators over a specific period and estimate its confidence interval. The formula is: EAPC = 100 × (exp^(β)^ − 1) [11].

#### 2.3.3 Average Annual Percentage Change

Average annual percentage change (AAPC) describes the average trend of health indicators over a longer period, being the weighted average of APCs for multiple periods. The formula is: AAPC = (∑_*i*_ *w*_*i*_ *β*_*i*_)/(∑_*i*_ *w*_*i*_) × 100 [11], where *βi* is the APC for the *i*‐th period, and *W*_*i*_ is the length of the period.

### 2.4 Data Analysis

Data analysis was conducted using R software (R version 4.4.0) and Joinpoint regression software (version 5.0.2). Initially, the IBD data from 1990 to 2021 underwent a series of preprocessing and standardization procedures. Subsequently, the ASR‐Incidence, ASR‐Prevalence, ASR‐Deaths, ASR‐DALYs, and EAPC were calculated. The Joinpoint regression model (JRM) was employed to analyze the time trends of IBD in various regions and calculate APC and AAPC [12]. The primary function of the JRM is to identify changes in time trends and evaluate the trend changes over different periods.

To predict the future trends of IBD for the next decade, we used the Nordpred package (version 4.4.0) of R software and the Bayesian age‐period‐cohort (BAPC) model. The Nordpred package estimates future incidence, prevalence, deaths, and DALYs by analyzing historical data and population projections. The BAPC model uses Bayesian methods to comprehensively analyze data from different age groups, periods, and birth cohorts, improving the accuracy of the prediction results [13].

### 2.5 Statistical Testing

All statistical analyses used two‐sided tests, with *p* < 0.05 considered statistically significant. To ensure the reliability of the results, 95% confidence intervals (CI) for the estimated parameters of the models were calculated, and multiple methods (BAPC model and INLA model) were used to verify the stability and consistency of the prediction results.

## 3 Results

### 3.1 Global, Different SDI Regions, and China's IBD Status in 2021

**FIGURE 1:**
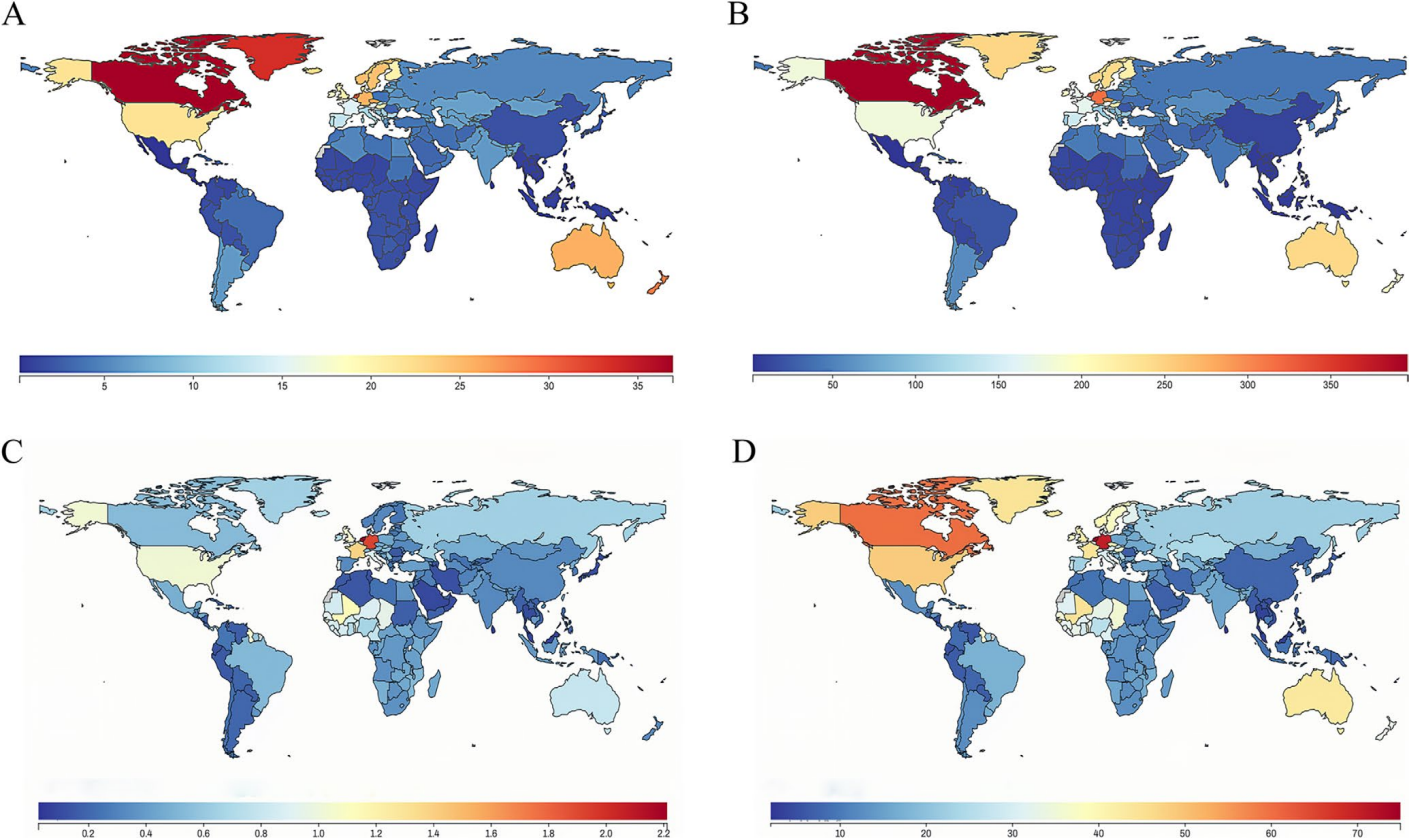
Global IBD epidemic status in 2021. (A) The global incidence of IBD in 2021; (B) The global prevalence of IBD in 2021; (C) The global death rate of IBD in 2021; (D) The global DALYs of IBD in 2021.

According to data provided by the GBD study, the incidence and prevalence of IBD are quantified across different countries. In 2021, three countries with the highest incidence rates are Canada [26.83 (23.30–30.76)], Greenland [24.57 (21.79–28.44)], and New Zealand [23.69 (20.89–27.88)], significantly higher than in China [1.40 (1.21–1.68)], while the three countries with the lowest incidence rates are Mexico [0.20 (0.17–0.25)], Philippines [0.53 (0.44–0.65)] and El Salvador [0.58 (0.50–0.71)]. Canada [325.64 (278.94–385.56)], Netherlands [278.48 (241.04–327.43)], and San Marino [269.34 (230.41–321.11)] are currently the countries with the highest prevalence, much higher than in China [9.16 (7.80–11.01)], while Mexico [1.86 (1.54–2.29)], Philippines [4.30 (3.59–5.24)] and Cambodia [4.57 (3.83–5.55)] are the lowest. The highest death rates were observed in Netherlands [2.21 (1.85–2.43)], Germany [1.92 (1.63–2.10)] and France[1.38 (1.17–1.54)], much higher than in China [0.33 (0.25–0.44)], while Singapore [0.03 (0.02–0.03)], Sri Lanka [0.03 (0.02–0.04)] and Guam [0.06 (0.05–0.10)] are the lowest. The highest DALYs were observed in Netherlands [74.96 (60.11–91.48)], Germany [70.06 (57.95–84.98)] and Canada [60.05 (43.45–78.87)], much higher than in China [7.68 (6.20–9.57)], while Singapore [1.97 (1.45–2.60)], Sri Lanka [2.05 (1.50–2.73)] and Malaysia [2.97 (2.38–3.73)] are the lowest (Figure 1A–D and Tables [Link], [Link]).

In 1990, the global IBD incidence rate was 4.22 (3.72–4.88), with the highest rates in high SDI (Socio‐Demographic Index) regions at 10.65 (9.47–12.26), far exceeding those in high‐middle, middle, low‐middle, low SDI regions, and China. By 2021, the global incidence rate had risen to 4.45 (3.87–5.19), still highest in high SDI regions. China's incidence rate, while significantly lower than these regions, nearly doubled from 1990 (change of 90.54%). Prevalence trends mirrored incidence trends. Regarding Deaths and DALYs, the global IBD Deaths and DALYs in 2021 decreased compared to 1990 (−15.38% and − 16.11%, respectively). In high SDI regions, Deaths increased slightly, and DALYs remained nearly constant (12.67%, 3.92%). In contrast, Deaths and DALYs decreased in high‐middle, middle SDI regions, and China, with China showing the largest decline in deaths and DALYs (−56.00%, −58.22%) (Table 1).

**TABLE 1:** Age‐standardized incidence, prevalence, deaths, and disability‐adjusted life years (DALYs) of IBD in the global and Chinese populations in 1990 and 2021.

Location	Incidence			Prevalence		
	ASR_1990	ASR_2021	Change	ASR_1990	ASR_2021	Change
Global	4.22 (3.72, 4.88)	4.45 (3.87, 5.19)	5.45%	48.02 (41.94, 55.78)	44.88 (38.80, 52.86)	−6.54%
High SDI	10.65 (9.47, 12.26)	11.59 (10.14, 13.38)	8.83%	129.54 (114.44, 147.63)	132.76 (115.02, 154.34)	2.49%
High‐middle SDI	3.00 (2.63, 3.5)	3.29 (2.84, 3.90)	9.67%	35.33 (30.64, 41.63)	31.63 (27.06, 37.88)	−10.47%
Middle SDI	1.57 (1.36, 1.89)	2.36 (2.04, 2.85)	50.32%	14.00 (11.87, 16.94)	19.59 (16.62, 23.77)	39.93%
Low‐middle SDI	3.62 (3.15, 4.33)	4.27 (3.70, 5.13)	17.96%	27.98 (23.72, 33.50)	32.46 (27.47, 39.12)	16.01%
Low SDI	2.54 (2.2, 3.07)	2.99 (2.59, 3.57)	17.72%	20.65 (17.46, 24.95)	22.84 (19.41, 27.61)	10.61%
China	0.74 (0.64, 0.9)	1.40 (1.21, 1.68)	90.54%	5.59 (4.73, 6.69)	9.16 (7.80, 11.01)	63.86%

Location	Deaths			DALYs		
	ASR_1990	ASR_2021	Change	ASR_1990	ASR_2021	Change
Global	0.60 (0.52, 0.66)	0.52 (0.46, 0.57)	−15.38%	21.54 (18.47, 24.82)	18.07 (15.67, 20.91)	−16.11%
High SDI	0.71 (0.65, 0.75)	0.80 (0.70, 0.86)	12.67%	33.68 (27.58, 41.18)	35.00 (28.33, 42.95)	3.92%
High‐middle SDI	0.53 (0.48, 0.58)	0.40 (0.35, 0.47)	−24.53%	19.30 (16.89, 22.30)	13.07 (11.20, 15.20)	−32.28%
Middle SDI	0.53 (0.35, 0.65)	0.33 (0.27, 0.39)	−37.74%	15.66 (11.69, 18.43)	10.92 (9.23, 12.63)	−30.27%
Low‐middle SDI	0.47 (0.33, 0.58)	0.38 (0.31, 0.47)	−19.15%	17.58 (13.67, 21.26)	15.37 (12.63, 18.66)	−12.57%
Low SDI	0.53 (0.37, 0.66)	0.47 (0.34, 0.58)	−11.32%	19.23 (14.31, 23.58)	17.98 (13.29, 21.83)	−6.50%
China	0.75 (0.47, 0.95)	0.33 (0.25, 0.44)	−56.00%	18.38 (12.69, 23.14)	7.68 (6.20, 9.57)	−58.22%

Abbreviation: ASR = age adjusted rates.

### 3.2 Changes in Global, Different SDI Regions, and China's IBD From 1990 to 2021

**FIGURE 2:**
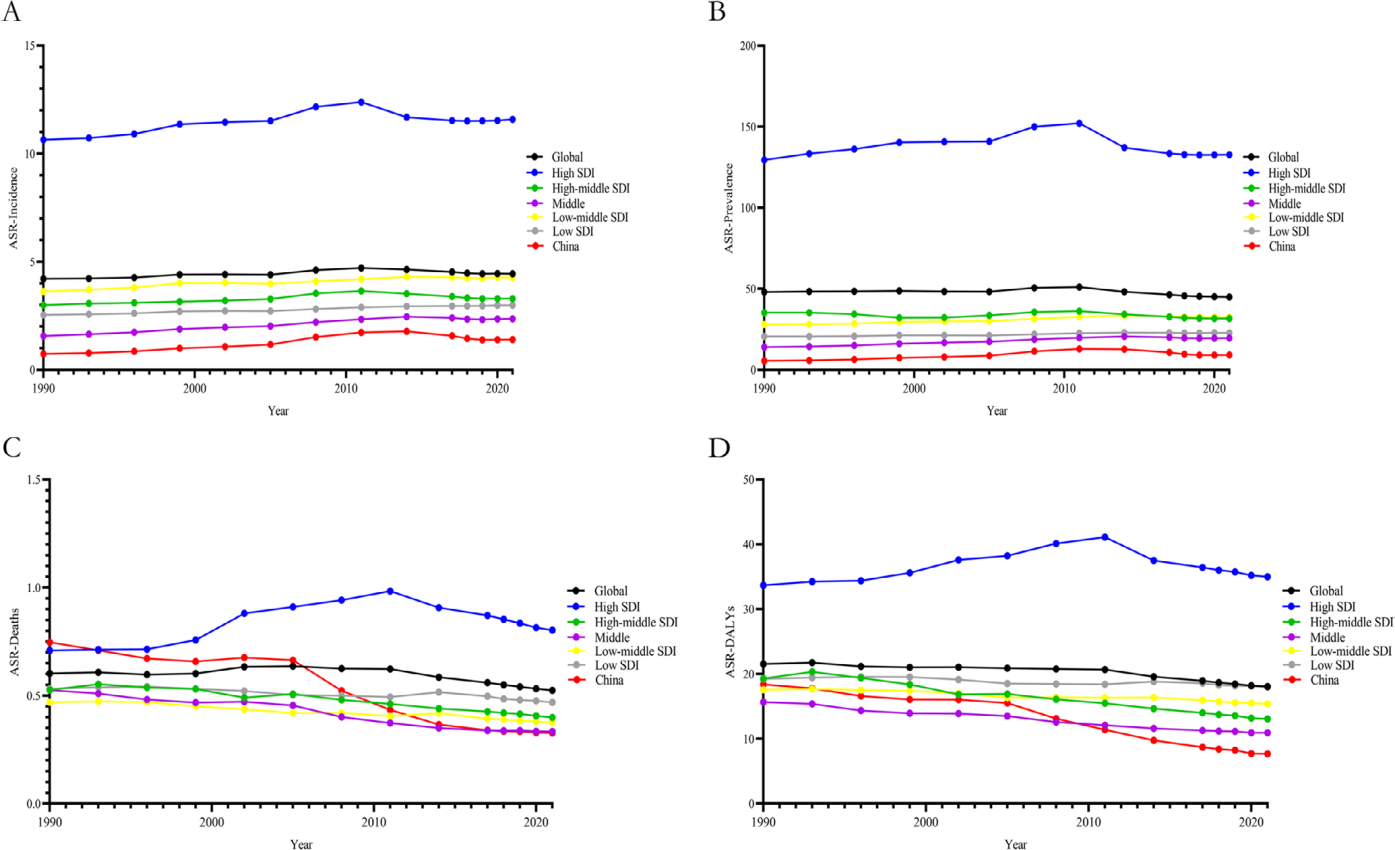
Changes in the epidemiological burden of IBD globally and in China from 1990 to 2021 (age‐standardized). (A) Changes in the incidence of IBD from 1990 to 2021; (B) Changes in the prevalence of IBD from 1990 to 2021; (C) Changes in the death rate of IBD from 1990 to 2021; (D) Changes in DALYs of IBD from 1990 to 2021.

From 1990 to 2021, high SDI regions experienced a rise and then a fall in incidence, prevalence, deaths, and DALYs around 2010, maintaining high stability overall. Similarly, other SDI regions and China saw increases in incidence and prevalence followed by declines around 2010, with continuous decreases in Deaths and DALYs, remaining much lower than high SDI regions (Figure 2A–D and Tables [Link], [Link]).

**FIGURE 3:**
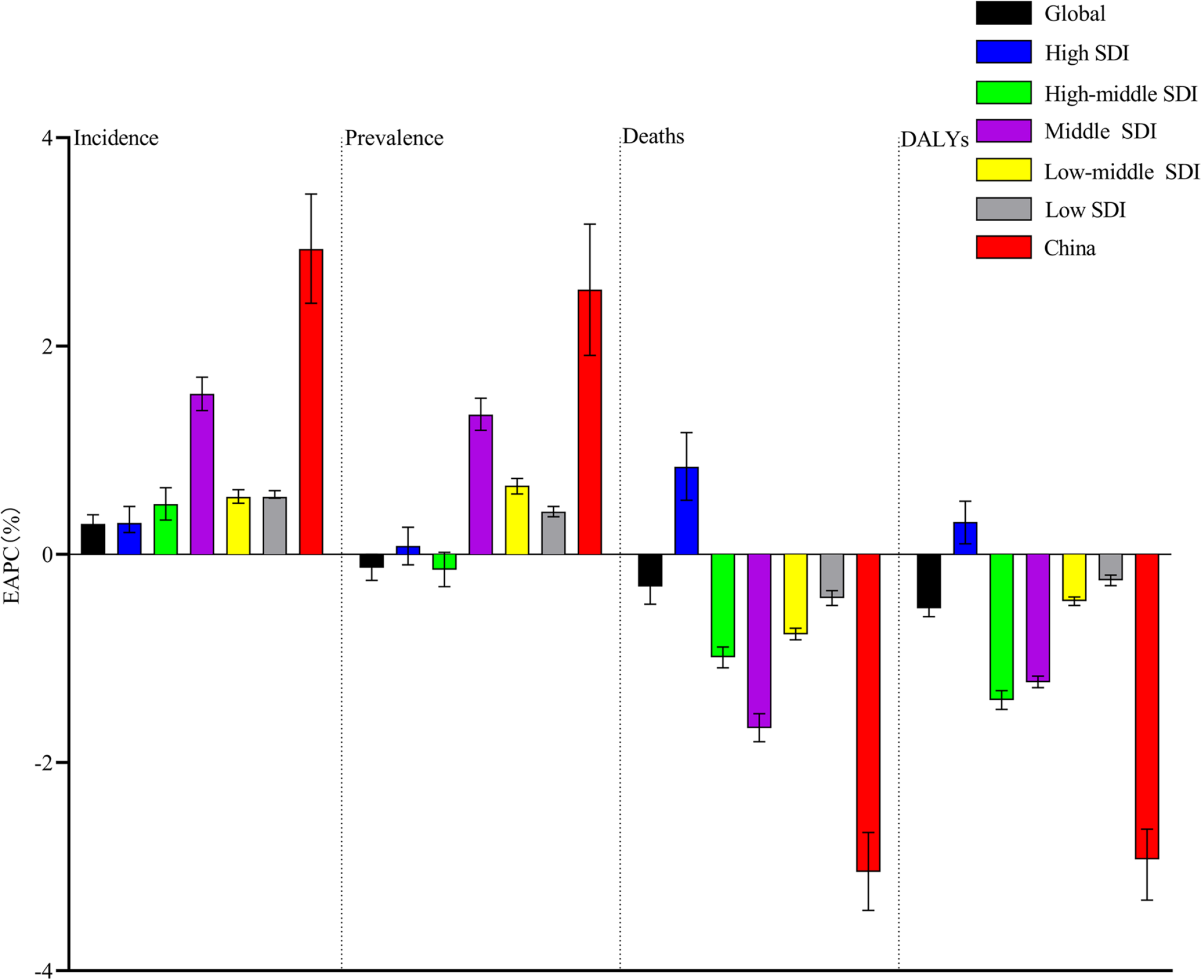
Estimated annual percentage change (EAPC) in the incidence, prevalence, deaths, and DALYs of IBD globally and in China from 1990 to 2021 (age‐standardized).

Quantifying these changes using EAPC values and confidence intervals, China showed the most significant changes in incidence, prevalence, Deaths, and DALYs, with the highest increases in incidence and prevalence and the greatest decreases in Deaths and DALYs. Middle SDI regions also saw notable increases in incidence and prevalence and significant decreases in Deaths and DALYs. High SDI regions showed minimal changes over the past 31 years (Figure 3).

### 3.3 Temporal Trends of IBD in Global, Different SDI Regions and Countries From 1990 to 2021

Using mathematical models to determine optimal points, the global, different SDI regions, and China's IBD trends were divided into four significant periods. Globally, from 1990 to 2001, the incidence rate of IBD increased significantly (APC = 0.527, *p* < 0.05). From 2001 to 2005, it decreased slightly but was not statistically significant (*p* > 0.05). From 2005 to 2010, it rose again significantly (APC = 1.575, *p* < 0.05), and from 2010 to 2021, it declined significantly (APC = −0.644, *p* < 0.05). Overall, the global incidence rate increased by 0.185% per year (*p* < 0.05). Similarly, global prevalence declined by −0.242% per year (*p* < 0.05), with a stable trend over the past 4 years (2018–2021) (*p* > 0.05). Global deaths and DALYs showed slow significant declines over the past 31 years (−0.488%, −0.592%) (*p* < 0.05).

In High SDI regions, prevalence and DALYs remained relatively unchanged (0.026%, 0.103%) (*p* > 0.05), while incidence and deaths increased slightly (0.282%, 0.388%) (*p* < 0.05). Low SDI regions continue to exhibit minimal fluctuations in their levels of increase or decrease. Regardless of the incidence, prevalence, mortality, or DALYs, the Middle SDI region exhibited a greater magnitude of change than the other SDI regions (Table 2).

**TABLE 2:** Annual percentage change (APC) in the age‐standardized incidence, prevalence, deaths, and DALYs of IBD in the global and different SDI regions from 1990 to 2021.

Catalog	Incidence		Prevalence		Deaths		DALYs	
	Endpoint	APC	Endpoint	APC	Endpoint	APC	Endpoint	APC
Global	1990–2001	0.527*	1990–2005	0.006	1990–1998	−0.283	1990–1994	0.184
	2001–2005	−0.185	2005–2010	1.340*	1998–2003	1.543*	1994–1997	−0.999
	2005–2010	1.575*	2010–2018	−1.581*	2003–2012	−0.518*	1997–2011	−0.151*
	2010–2021	−0.644*	2018–2021	−0.503	2012–2021	−1.751*	2011–2021	−1.393*
AAPC	0.185*		−0.242*		−0.488*		−0.592*	
High SDI	1990–2006	0.633*	1990–2006	0.564*	1990–1998	0.250	1990–1996	0.355
	2006–2010	1.766*	2006–2010	2.203*	1998–2002	5.314*	1996–2011	1.255*
	2010–2015	−1.671*	2010–2015	−2.927*	2002–2012	0.901*	2011–2014	−3.141*
	2015–2021	0.101	2015–2021	−0.228	2012–2021	−2.163*	2014–2021	−1.066*
AAPC	0.282*		0.026		0.388*		0.103	
High‐middle SDI	1990–2005	0.536*	1990–1995	−0.238	1990–1994	1.901*	1990–1994	1.707*
	2005–2010	2.573*	1995–2000	−2.184*	1994–2001	−1.817*	1994–2001	−2.617*
	2010–2019	−1.251*	2000–2010	1.534*	2001–2004	0.900	2001–2005	−0.283
	2019–2021	−0.285	2010–2021	−1.462*	2004–2021	−1.345*	2005–2021	−1.536*
AAPC	0.285*		−0.425*		−0.823*		−1.208*	
Middle SDI	1990–2001	2.055*	1990–2006	1.578*	1990–1998	−1.544*	1990–1998	−1.620*
	2001–2004	1.001	2006–2009	2.683	1998–2003	0.134	1998–2003	−0.173
	2004–2014	2.183*	2009–2014	1.460*	2003–2013	−2.930*	2003–2012	−1.754*
	2014–2021	−0.990*	2014–2021	−1.008*	2013–2021	−0.687*	2012–2021	−0.919*
AAPC	1.299*		1.075*		−1.506*		−1.224*	
Low‐middle SDI	1990–2000	1.162*	1990–2006	0.591*	1990–1994	0.277	1990–2000	−0.173*
	2000–2005	−0.214	2006–2009	1.679	1994–2011	−0.961*	2000–2005	−1.166*
	2005–2014	0.870*	2009–2014	0.973*	2011–2014	0.734	2005–2014	−0.057
	2014–2021	−0.182	2014–2021	−0.551*	2014–2021	−1.390*	2014–2021	−0.883*
AAPC	0.550*		0.498*		−0.736*		−0.461*	
Low SDI	1990–2001	0.699*	1990–2001	0.358*	1990–1995	0.585*	1990–1999	0.211*
	2001–2005	−0.087*	2001–2005	−0.218	1995–2011	−0.711*	1999–2006	−0.962*
	2005–2011	1.161*	2005–2012	1.135*	2011–2014	1.873*	2006–2015	0.20*
	2011–2021	0.273*	2012–2021	−0.042	2014–2021	−1.400*	2015–2021	−0.668*
AAPC	0.549*		0.342*		−0.412*		−0.228*	

* *p* < 0.05.

### 3.4 The Correlation Between Age and Gender in Relation to the Development of IBD Across Different Countries

The findings of the present study demonstrate the potential impact of varying SDI levels on the development of IBD. The investigation further extends to the country level by selecting two High SDI countries (Germany and Canada), two Low SDI countries (Somalia and Yemen), and nine newly industrialized countries (China, Argentina, Colombia, India, Iran, Russian, Thailand and Malaysia). The analysis incorporates age, gender, and temporal differences in the development of IBD between 1990 and 2021. Average trends by age, gender, and time period are expressed as AAPC values.

**FIGURE 4:**
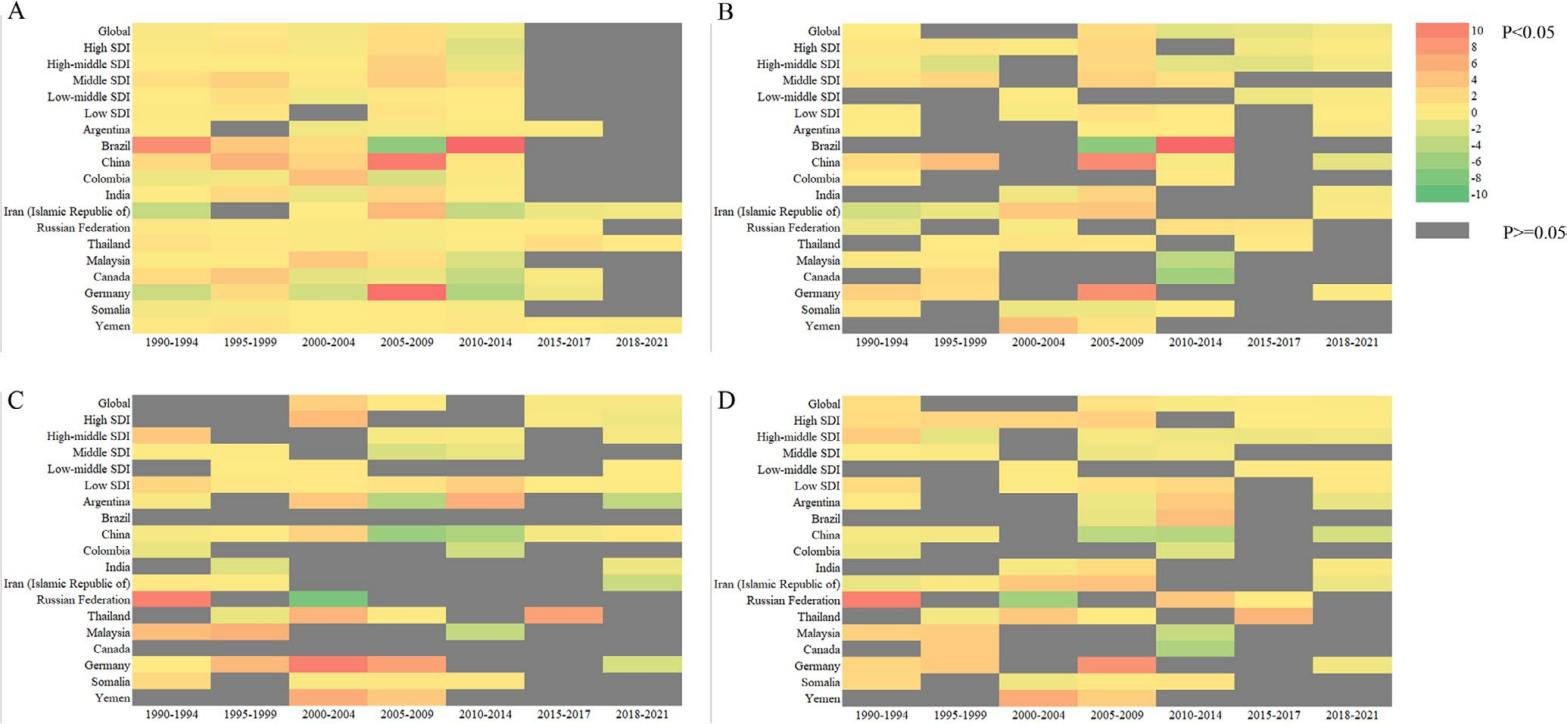
Changes in the incidence, prevalence, death rate, and DALYs of IBD by age and sex between 1990 and 2021 at the national level (the data are presented in the form of a heat map with AAPC values). (A) Changes of incidence; (B) Changes of prevalence; (C) Changes of death rate; (D) Changes of DALYs.

Among the countries included in the study, the change in the incidence of IBD between 1990 and 2021 did not demonstrate significant disparities in age or gender. The most substantial increases in IBD incidence rate were observed in China and Malaysia. In China, the rise in IBD prevalence is predominantly among individuals aged 20 to 60 years, while in Malaysia, it is primarily among those under 50 years of age. Significant declines in IBD‐related mortality have been observed in China, India, and Malaysia, particularly among younger age groups. Notably, females in China have exhibited a higher rate of decline compared to males, while males in Malaysia have shown a higher rate of decline than females. In contrast, Germany has experienced a substantial increase in IBD‐related death rates over the past 31 years, with the majority of cases occurring among individuals over 50 years of age. Yemen has exhibited a sustained high level of increase in IBD‐related mortality across all age groups. A substantial decrease in DALYs was observed in China and Russia. In China, the majority of DALYs were attributed to individuals over the age of 55, while in Russia, the majority of DALYs were attributed to individuals under the age of 55. Iran, Thailand, and Canada experienced minor increases in DALYs (Figure 4).

### 3.5 Examination of the Temporal Progression of IBD Development Across Various Nations

**FIGURE 5:**
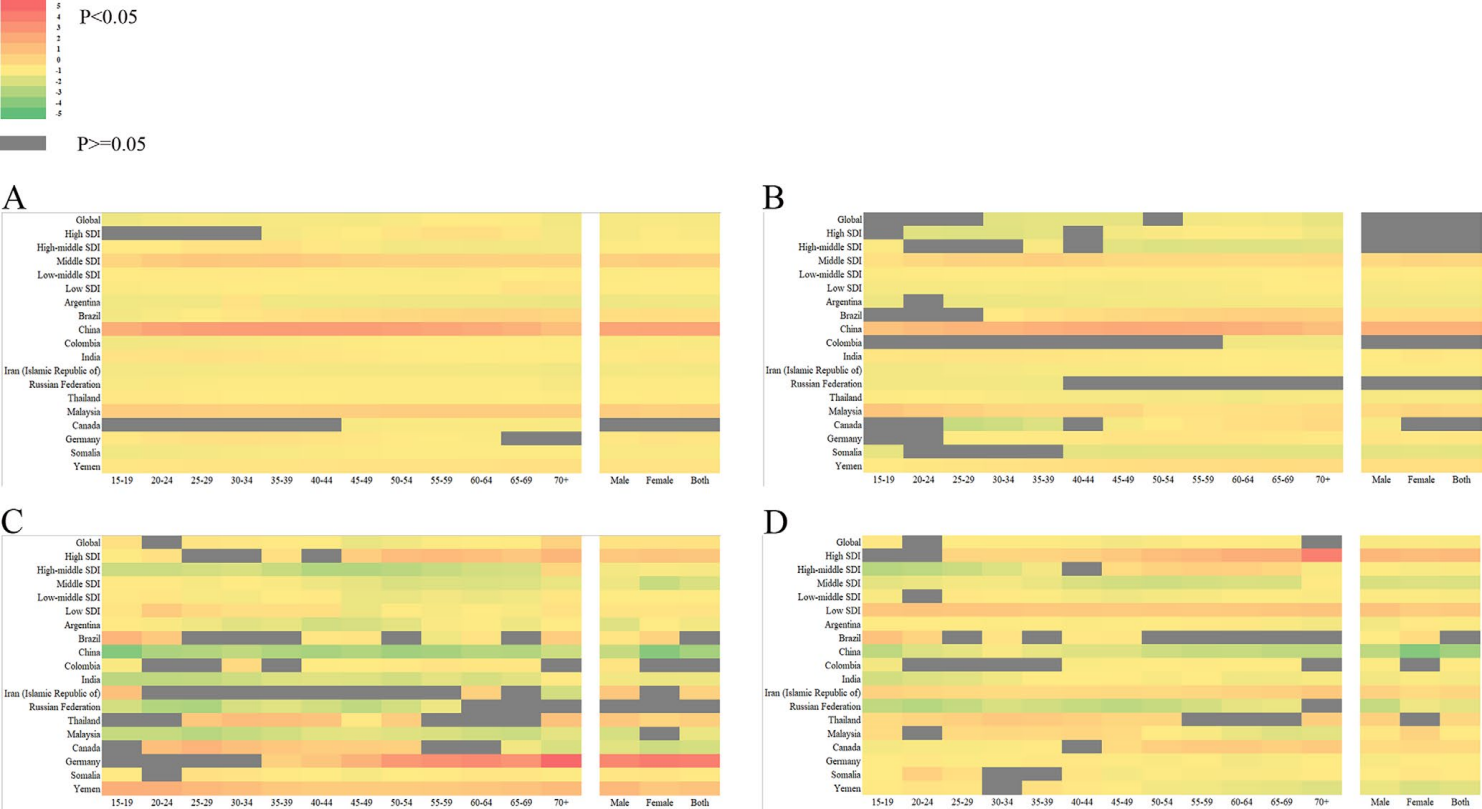
Changes in the incidence, prevalence, death rate, and DALYs of IBD by time between 1990 and 2021 at the national level. (A) Changes of incidence; (B) Changes of prevalence; (C) Changes of death rate; (D) Changes of DALYs.

The temporal trends in the incidence and prevalence of IBD in the countries under consideration, as well as within the specified time frames, are predominantly influenced by the period up to the year 2015. A notable increase in IBD incidence and prevalence was observed between the years 2005 and 2009 in China and Germany, and between 2010 and 2014 in Brazil. However, analyses of IBD‐related death rates and DALYs at the national level revealed no significant patterns, with variations observed across different countries. In China, a notable decline in IBD‐related death rates and DALYs was observed between 2005 and 2014, while in Russia, a significant decrease was recorded between 2000 and 2004 (Figure 5).

### 3.6 Predictions for IBD Development in Global and Chinese Populations Aged 15–49 Over the Next Decade

**FIGURE 6:**
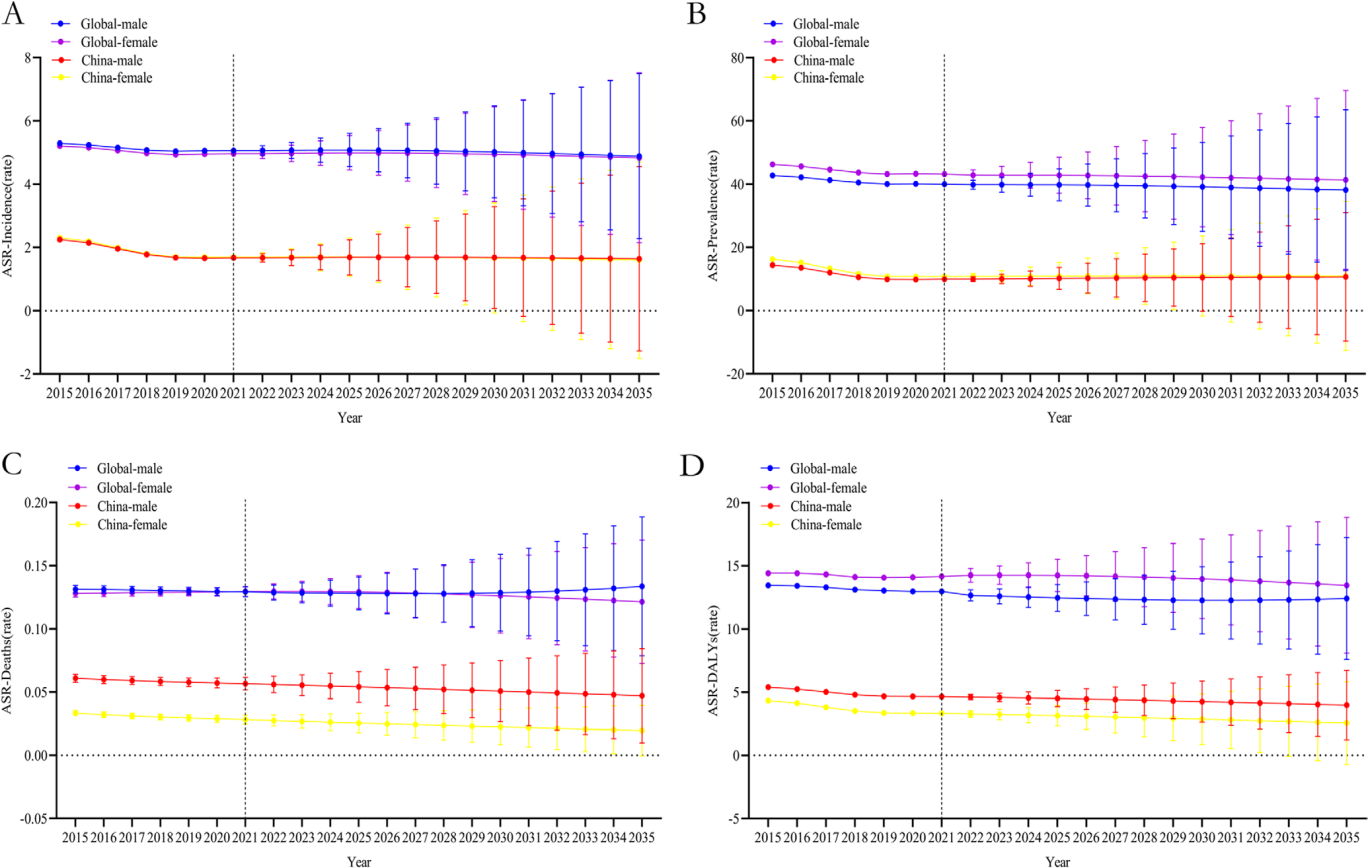
Projected changes in the epidemiological burden of IBD in the 15–49 age group globally and in China from 2022 to 2035. (A) Projected changes in the incidence of IBD in the 15–49 age group globally and in China from 2022 to 2035; (B) Projected changes in the prevalence of IBD in the 15–49 age group globally and in China from 2022 to 2035; (C) Projected changes in the deaths rate of IBD in the 15–49 age group globally and in China from 2022 to 2035; (D) Projected changes in disability‐adjusted life years (DALYs) due to IBD in the 15–49 age group globally and in China from 2022 to 2035.

From an incidence perspective, in the global 15–49 age group, the 2021 male incidence rate was 5.06 (5.03, 5.09) and the female rate was 4.96 (4.93, 4.99). By 2035, these rates are expected to be 4.88 (2.28, 7.49) for males and 4.83 (2.15, 7.52) for females, maintaining stability from 2021 to 2035. In China, the 2021 male incidence rate was 1.67 (1.63, 1.71) and the female rate was 1.69 (1.65, 1.74). By 2035, these rates are expected to be 1.64 (−1.27, 4.55) for males and 1.60 (−1.50, 4.70) for females, also maintaining stability. China's rates are lower than the global average (Figure 6A and Table S10).

In terms of prevalence, the global 15–49 age group had a male prevalence rate of 39.98 (39.89, 40.06) and a female rate of 43.17 (43.08, 43.26) in 2021. By 2035, these rates are expected to be 38.12 (12.75, 63.51) for males and 41.35 (13.10, 69.60) for females, maintaining stability. In China, the 2021 male prevalence rate was 9.93 (9.83, 10.04) and the female rate was 10.80 (10.69, 10.91). By 2035, these rates are expected to be 10.68 (−9.65, 31.01) for males and 10.93 (−12.61, 34.48) for females, remaining stable. China's rates are lower than the global average (Figure 6B and Table S11).

For deaths rate, the global 15–49 age group had a male Deaths rate of 0.13 (0.12, 0.13) and a female rate of 0.13 (0.12, 0.13) in 2021. By 2035, these rates are expected to be 0.13 (0.08, 0.19) for males and 0.12 (0.07, 0.17) for females, maintaining stability. In China, the 2021 male Deaths rate was 0.06 (0.05, 0.06) and the female rate was 0.03 (0.02, 0.03). By 2035, these rates are expected to be 0.05 (0.01, 0.08) for males and 0.02 (−0.00, 0.04) for females, maintaining stability. China's rates are lower than the global average (Figure 6C and Table S12).

In terms of DALYs, the global 15–49 age group had male DALYs of 12.97 (12.92, 13.02) and female DALYs of 14.16 (14.11, 14.21) in 2021. By 2035, these rates are expected to be 12.41 (7.58, 17.24) for males and 13.46 (8.08, 18.84) for females, maintaining stability. In China, the 2021 male DALYs were 4.65 (4.58, 4.72) and female DALYs were 3.32 (3.26, 3.38). By 2035, these rates are expected to be 3.39 (1.23, 7.73) for males and 2.56 (−0.71, 5.84) for females, maintaining stability. China's rates are lower than the global average (Figure 6D and Table S13).

## 4 Discussion

This study presents the current disease status and the development of IBD based on the most recent data released by the GBD database in May 2021. Given the potential for substantial economic, cultural, and climatic variations across the various countries encompassed within the designated geographic regions on a global scale, this study employs the degree of social development as the primary criterion for the delineation of regions. The study undertakes a comprehensive description of the global landscape, delineating the various SDI regions (High, High‐middle, Middle, Low‐middle, and Low SDI). The study describes the changes and characteristics of IBD incidence, prevalence, death rate, and DALYs in the world, in selected newly industrialized countries (Argentina, Brazil, Colombia, China, India, Iran, Russia, Malaysia, and Thailand), in the two most developed countries in the world (Canada and Germany), and in the two least developed countries in the world (Somalia and Yemen).

On a global scale, the 31‐year period from 1990 to 2021 was characterized by accelerated population and economic growth, with national and regional economic development driving advancements in social and medical care, heightened disease awareness, and a substantial rise in life expectancy [14]. During the late 19th and early 20th centuries, there was a notable increase in the prevalence of IBD patients in Europe and the United States. This phenomenon occurred concurrently with the prevailing perception of IBD as a rare disease in China [15]. The epidemiological evolution of IBD can be categorized into the following stages: emergence, accelerated incidence, compound prevalence, and prevalence equilibrium. Developing countries are in the emergence stage, while newly industrialized countries with rapid economic development are in the accelerated incidence stage. In contrast, developed countries in the West have mostly reached the compound prevalence stage and are gradually progressing toward the prevalence equilibrium stage [16]. Although these newly industrialized countries have seen an increase in the number of patients with IBD along with rapid economic development, a much larger population is found in developing countries with relatively slow economic development. The increasing availability of medications, including traditional hormones, immunosuppressants, and biologics (vedolizumab, infliximab), has greatly reduced the number of deaths from IBD, especially in more economically developed countries and regions, where scientific and effective management can ensure that patients with IBD can achieve long‐term survival with the disease [17]. The DALY concept was developed to quantify the total number of healthy life‐years lost by a patient. In the context of chronic disease management, higher values indicate superior management of the disease. However, as a country or region grapples with a surge in new cases of disease, the DALY value declines. Conversely, as the incidence rate of a given disease stabilizes and the medical investment can gradually cover the entire population, the patient's life expectancy with the disease continues to lengthen, and the value of DALYs continues to increase [18]. China's economic development over the past 30–40 years has been significant, and while this social development has led to enhanced medical care, it has also influenced the country's living and eating habits. The advent of a large number of new‐onset IBD patients can be attributed to the following factors: high work pressure, lack of sleep, high sugar and high fat diets, and the use of various types of food additives [19]. A similar trend has been observed in other newly industrialized countries with rapid economic development, such as Brazil, India, Colombia, and Malaysia, which exhibit characteristics of IBD development analogous to those of China. The majority of these countries are classified within the high‐middle, middle, and low‐middle SDI regions, exhibiting a certain degree of domestic economic disparity between the affluent and the impoverished. Projections indicate an escalation in the number of new IBD cases in the future. As international exchanges in these regions have deepened, advanced IBD management concepts have gradually been introduced, resulting in a decline in IBD‐related death rates and DALYs. In the context of global least developed countries (Low SDI), such as Yemen and Somalia, despite the substantial increase in population, the absence of comprehensive economic development and medical infrastructure hinders the diagnosis, treatment, and counting of IBD patients. Over the past three to four decades, the incidence, prevalence, death rates, and DALYs of IBD have remained at a low level, independent of genetic, climatic, dietary, and other factors, and there has been no significant evolution of IBD.

While the majority of High SDI countries exhibit comparable patterns of IBD evolution, notable variations among nations are evident. As presented in this paper, the increase in IBD‐related deaths in Germany is particularly pronounced in the period 1990–2021, and similarly, Western European countries such as the United Kingdom and the Netherlands have significantly higher IBD‐related deaths in 2021 than Canada, the United States, and Japan, which are also in the High SDI camp. A prospective cohort study of 5763 IBD patients in the United Kingdom demonstrated that IBD patients who consumed processed meat products more than four times per week exhibited a significantly higher death rate compared to those who consumed them 0.1–0.9 times per week (HR = 1.53, 95% CI: 1.06–2.23) [20]. Western dietary patterns have been identified as a significant contributing factor to the onset of IBD [21]. The influence of Western dietary patterns on national and regional economic development is multifaceted and varies across different regions. However, it is postulated that the proliferation of Western dietary habits may play a pivotal role in the accelerated growth of IBD cases observed in recently industrialized nations. The reduction in the diversity of gut flora has been linked to chronic inflammation, which, in turn, has been associated with the development of immune and metabolic diseases [22]. Malaysia and China have demonstrated a high degree of similarity with regard to economic, geopolitical, cultural, living, and dietary habits. Consequently, the development of IBD in these two countries is characterized by notable parallels. The inherent complexity of IBD necessitates a substantial investment in healthcare to enhance the scientific management of the condition. IBD, with the United States allocating 17% of its GDP to healthcare, Japan dedicating 11%, China 7%, and the major European countries investing 10% or more. Notably, India, a similarly populous nation, invests less than 5% [23]. This disparity in healthcare investment may be a contributing factor to the less pronounced evolution of IBD in India compared to China. The presence of a congruence between countries and regions exhibiting analogous SDI levels has been observed to coincide with the manifestation of IBD trends that bear a resemblance to one another. However, it is imperative to acknowledge that disparities in lifestyle patterns, which are distinct from one country to another, have the potential to exert an influence on the progression of IBD within the context of a particular nation. Epidemiological studies have demonstrated that during early childhood, boys exhibit a higher risk of developing CD compared to girls. Additionally, girls demonstrate a significantly elevated risk of CD after puberty. The gender disparities in IBD may be influenced by geographic location or other environmental factors. For instance, the prevalence and severity of CD are higher in women than in men in Western populations. Moreover, female patients report poor quality of life, severe disability, and major depression more frequently than their male counterparts. However, this pattern is not observed in Asian populations [24]. The findings of this study indicate that there is no longer a significant gender disparity in the global burden of IBD. China appears to exhibit a more pronounced decline in the IBD‐related death rate and DALYs among females. In Brazil, the increase in the IBD‐related death rate was higher among females than among males. In Thailand, the increase in the IBD‐related death rate was higher among males than among females. The decrease in the IBD‐related death rate was higher among females than among males in the Middle SDI regions. It is important to acknowledge that the air pollution issues that are pervasive in newly industrialized countries can impede IBD patients' ability to maintain a stable disease state. A prospective cohort study demonstrated that prolonged exposure to PM_2.5_, PM_10_, and NO_2_ was associated with an elevated surgical resection rate and an increased risk of all‐cause death rate in patients with IBD [25].

From a temporal perspective, the years 1990 to 2021 were segmented into seven distinct time periods, during which the progression of IBD exhibited a discernible temporal correlation. Within these delineated periods, China and Germany demonstrated the most accelerated growth in IBD incidence and prevalence during the years 2005–2009, while Brazil exhibited a substantial surge in IBD incidence and prevalence during the period of 2010–2014. Subsequent to 2015, the majority of countries and regions worldwide exhibited no substantial alterations in IBD incidence and prevalence. However, fluctuations in death rates and DALYs were observed, with notable declines in IBD death rates and DALYs recorded in China from 2005 to 2014 and in Russia from 2000 to 2004. This phenomenon may be closely related to the increased national emphasis on IBD and the clinical use of emerging treatment concepts and drugs. For example, infliximab was introduced to China in 2007.

The increased utilization of biologics has led to a substantial improvement in the prognosis of patients diagnosed with IBD, as well as the significant economic implications associated with their use [26]. In 2020, the mean yearly direct expenses in the European region will amount to €3524 for CD patients and €2088 for UC patients. Among the five‐year expenditures, 73% and 48%, respectively, are attributable to biologics for both CD and UC patients [27]. In the 2018–2019 period, the economic burden of IBD patients in China is primarily evident in two aspects: elevated medical expenditures and diminished income. The average annual cost of medical expenditures is estimated to be approximately $11, 668.68 yuan. Moreover, 49.50% of patients have a monthly income of less than 5000 yuan, and 63.50% are unable to work full‐time. It is noteworthy that only approximately 26.60% of IBD patients receive more than three biologics treatments per year, and among the IBD patients who receive regular biologics treatments, 61.70% need to bear the cost out of their own pocket [28]. In some underdeveloped regions, such as in 2019, only 6.3% of IBD patients in Colombia receive treatment with biologics due to government regulation of high‐priced medications, predominantly with adalimumab, at an average cost of $18 428. This cost is substantially lower than the costs observed in the United States ($36 051) and Canada ($28 773). Nevertheless, the treatment of IBD patients is to some extent limited by the lack of medical resources [29].

A prediction of the development of IBD in the world and China in the years 2022–2035 is made. For individuals between the ages of 15 and 49 (the demographic with the highest incidence of IBD), the incidence, prevalence, death rate, and DALYs of IBD are expected to remain relatively stable on a global scale. In China, between the ages of 15 and 49, the incidence and prevalence of IBD are projected to remain consistent with current standards, while the death rate and DALYs are predicted to exhibit a sustained decline. The incidence and prevalence of IBD in China are expected to remain consistent over the next decade, while the death rate and DALYs are projected to decline gradually. Given China's substantial population base, the number of new IBD patients is expected to remain high, and IBD research in China continues to face significant challenges.

This study mines the GDB2021 database to show the characteristics of IBD development globally, in different SDI regions, and in selected countries between 1990 and 2021. Against the background of relatively stable global IBD development, high SDI regions maintained high levels of incidence, prevalence, mortality, and DALYs; low SDI regions maintained low levels of IBD development; and newly industrialized countries, including China, experienced rapid development of IBD over the past 30–40 years, and it has become a nationwide public health problem. In the next decade, the incidence and prevalence of IBD in China will generally remain at the current level, and the death rate and DALYs caused by IBD will continue to decrease as research breakthroughs continue to be made. However, due to the large population size, IBD clinicians and researchers in China still face significant challenges.

## 5 Conclusion

In summary, this study reveals significant changes in the global and Chinese burden of IBD and predicts trends for the next 10 years. Although IBD incidence and prevalence rates have increased significantly in emerging industrialized countries like China, effective medical interventions and public health measures have significantly reduced IBD death rates and DALYs. Future research should continue to focus on IBD pathogenesis and epidemiology to provide scientific support for more effective public health policies and management strategies. By integrating multiple factors and adopting comprehensive control measures, the disease burden of IBD can be further reduced, improving the quality of life for patients.

## Ethics Statement

This study did not require ethical approval as it used publicly available data from the GBD database. All data were de‐identified and aggregated, ensuring no risk to individual privacy. The study was conducted in accordance with the Declaration of Helsinki and adhered to ethical guidelines for epidemiological research.

## Conflicts of Interest

The authors declare no conflicts of interest.

## Supporting information


**Table S1.** SDI values at the global, regional and national level.

**Table S2.** Raw data on IBD incidence in 2021 from GBD 2021 for 204 countries worldwide (Age‐standardized).

**Table S3.** Raw data on IBD prevalence in 2021 from GBD 2021 for 204 countries worldwide (Age‐standardized).

**Table S4.** Raw data on IBD deaths in 2021 from GBD 2021 for 204 countries worldwide (Age‐standardized).

**Table S5.** Raw data on IBD DALYs in 2021 from GBD 2021 for 204 countries worldwide (Age‐standardized).

**Table S6.** Raw data on IBD incidence from 1990 to 2021 for the world, different SDI regions and China (Age‐standardized).

**Table S7.** Raw data on IBD prevalence from 1990 to 2021 for the world, different SDI regions and China (Age‐standardized).

**Table S8.** Raw data on IBD deaths from 1990 to 2021 for the world, different SDI regions and China (Age‐standardized).

**Table S9.** Raw data on IBD DALYs from 1990 to 2021 for the world, different SDI regions and China (Age‐standardized).

**Table S10.** Raw data for predicting the trend of IBD incidence in 15‐49 year olds of global and Chinese population over the next decade (Age‐standardized).

**Table S11.** Raw data for predicting the trend of IBD prevalence in 15‐49 year olds of global and Chinese population over the next decade (Age‐standardized).

**Table S12.** Raw data for predicting the trend of IBD deaths in 15‐49 year olds of global and Chinese population over the next decade (Age‐standardized).

**Table S13.** Raw data for predicting the trend of IBD DALYs in 15‐49 year olds of global and Chinese population over the next decade (Age‐standardized).

## Data Availability

The data that support the findings of this study are available from the Global Burden of Disease (GBD) database. Restrictions apply to the availability of these data, which were used under license for this study. Data are available from the authors with the permission of the GBD database administrators.
