# Supplementary material for: Global and Chinese Burden of Inflammatory Bowel Disease From 1990 to 2021: A Systematic Analysis and Prediction of Disease Burden
Source: JGH Open. 2025 May 8;9(5):e70160. doi: 10.1002/jgh3.70160 (PMC12059552; doi:10.1002/jgh3.70160)
Supplement: Supplementary file 1 — Table S1. SDI values at the global, regional and national level. [file JGH3-9-e70160-s006.docx]

**Supplementary Table1. SDI values at the global, regional and national level**

| **Location** | **SDI value** | **Classification** |
| --- | --- | --- |
| Global | 0.65 | / |
| **Australasia** | 0.87 | High SDI |
| Australia | 0.87 | High SDI |
| New Zealand | 0.84 | High SDI |
| **High-income North America** | 0.87 | High SDI |
| Canada | 0.88 | High SDI |
| United States | 0.87 | High SDI |
| Greenland | 0.76 | High-middle SDI |
| **High-income Asia Pacific** | 0.87 | High SDI |
| Brunei | 0.86 | High SDI |
| Japan | 0.87 | High SDI |
| South Korea | 0.87 | High SDI |
| Singapore | 0.87 | High SDI |
| **Southern Latin America** | 0.72 | High-middle SDI |
| Argentina | 0.71 | High-middle SDI |
| Chile | 0.75 | High-middle SDI |
| Uruguay | 0.71 | High-middle SDI |
| **Western Europe** | 0.86 | High SDI |
| Andorra | 0.9 | High SDI |
| Austria | 0.87 | High SDI |
| Belgium | 0.89 | High SDI |
| Cyprus | 0.86 | High SDI |
| Denmark | 0.92 | High SDI |
| Finland | 0.89 | High SDI |
| France | 0.86 | High SDI |
| Germany | 0.87 | High SDI |
| Greece | 0.82 | High SDI |
| Iceland | 0.91 | High SDI |
| Ireland | 0.88 | High SDI |
| Israel | 0.82 | High SDI |
| Italy | 0.84 | High SDI |
| Luxembourg | 0.92 | High SDI |
| Malta | 0.84 | High SDI |
| Netherlands | 0.91 | High SDI |
| Norway | 0.91 | High SDI |
| Portugal | 0.78 | High-middle SDI |
| Spain | 0.82 | High SDI |
| Sweden | 0.88 | High SDI |
| Switzerland | 0.89 | High SDI |
| United Kingdom | 0.84 | High SDI |
| **Central Europe** | 0.81 | High SDI |
| Albania | 0.68 | Middle SDI |
| Bosnia and Herzegovina | 0.71 | High-middle SDI |
| Bulgaria | 0.79 | High-middle SDI |
| Croatia | 0.82 | High SDI |
| Czech Republic | 0.85 | High SDI |
| Hungary | 0.82 | High SDI |
| Macedonia | 0.75 | High-middle SDI |
| Montenegro | 0.79 | High-middle SDI |
| Poland | 0.84 | High SDI |
| Romania | 0.78 | High-middle SDI |
| Serbia | 0.75 | High-middle SDI |
| Slovakia | 0.84 | High SDI |
| Slovenia | 0.86 | High SDI |
| **Eastern Europe** | 0.79 | High-middle SDI |
| Belarus | 0.77 | High-middle SDI |
| Estonia | 0.86 | High SDI |
| Latvia | 0.83 | High SDI |
| Lithuania | 0.84 | High SDI |
| Moldova | 0.68 | Middle SDI |
| Russian Federation | 0.79 | High-middle SDI |
| Ukraine | 0.74 | High-middle SDI |
| **Central Asia** | 0.67 | Middle SDI |
| Armenia | 0.7 | High-middle SDI |
| Azerbaijan | 0.7 | High-middle SDI |
| Georgia | 0.7 | High-middle SDI |
| Kazakhstan | 0.74 | High-middle SDI |
| Kyrgyzstan | 0.61 | Middle SDI |
| Mongolia | 0.66 | Middle SDI |
| Tajikistan | 0.52 | Low-middle SDI |
| Turkmenistan | 0.7 | High-middle SDI |
| Uzbekistan | 0.63 | Middle SDI |
| **Southeast Asia** | 0.64 | Middle SDI |
| Cambodia | 0.48 | Low-middle SDI |
| Indonesia | 0.65 | Middle SDI |
| Laos | 0.52 | Low-middle SDI |
| Malaysia | 0.76 | High-middle SDI |
| Maldives | 0.66 | Middle SDI |
| Myanmar | 0.56 | Low-middle SDI |
| Philippines | 0.62 | Middle SDI |
| Sri Lanka | 0.68 | Middle SDI |
| Thailand | 0.68 | Middle SDI |
| Timor-Leste | 0.5 | Low-middle SDI |
| Vietnam | 0.61 | Middle SDI |
| Mauritius | 0.72 | High-middle SDI |
| Seychelles | 0.69 | High-middle SDI |
| **East Asia** | 0.71 | High-middle SDI |
| China | 0.71 | High-middle SDI |
| North Korea | 0.54 | Low-middle SDI |
| Taiwan | 0.86 | High SDI |
| **Oceania** | 0.47 | Low-middle SDI |
| Fiji | 0.64 | Middle SDI |
| Kiribati | 0.43 | Low SDI |
| Marshall Islands | 0.55 | Low-middle SDI |
| Federated States of Micronesia | 0.58 | Low-middle SDI |
| Papua New Guinea | 0.42 | Low SDI |
| Samoa | 0.58 | Low-middle SDI |
| Solomon Islands | 0.43 | Low SDI |
| Tonga | 0.62 | Middle SDI |
| Vanuatu | 0.48 | Low-middle SDI |
| American Samoa | 0.7 | High-middle SDI |
| Guam | 0.79 | High-middle SDI |
| Northern Mariana Islands | 0.76 | High-middle SDI |
| **South Asia** | 0.53 | Low-middle SDI |
| Bangladesh | 0.46 | Low-middle SDI |
| Bhutan | 0.57 | Low-middle SDI |
| India | 0.55 | Low-middle SDI |
| Nepal | 0.43 | Low SDI |
| Pakistan | 0.49 | Low-middle SDI |
| **Andean Latin America** | 0.63 | Middle SDI |
| Bolivia | 0.59 | Low-middle SDI |
| Ecuador | 0.64 | Middle SDI |
| Peru | 0.64 | Middle SDI |
| **Caribbean** | 0.64 | Middle SDI |
| Antigua and Barbuda | 0.72 | High-middle SDI |
| The Bahamas | 0.76 | High-middle SDI |
| Barbados | 0.74 | High-middle SDI |
| Belize | 0.6 | Low-middle SDI |
| Cuba | 0.69 | High-middle SDI |
| Dominica | 0.69 | High-middle SDI |
| Dominican Republic | 0.59 | Low-middle SDI |
| Grenada | 0.64 | Middle SDI |
| Guyana | 0.58 | Low-middle SDI |
| Haiti | 0.44 | Low SDI |
| Jamaica | 0.68 | Middle SDI |
| Saint Lucia | 0.65 | Middle SDI |
| Saint Vincent and the Grenadines | 0.61 | Middle SDI |
| Suriname | 0.64 | Middle SDI |
| Trinidad and Tobago | 0.7 | High-middle SDI |
| Bermuda | 0.81 | High SDI |
| Puerto Rico | 0.81 | High SDI |
| Virgin Islands, U.S. | 0.81 | High SDI |
| **Central Latin America** | 0.62 | Middle SDI |
| Colombia | 0.63 | Middle SDI |
| Costa Rica | 0.66 | Middle SDI |
| El Salvador | 0.59 | Low-middle SDI |
| Guatemala | 0.52 | Low-middle SDI |
| Honduras | 0.51 | Low-middle SDI |
| Mexico | 0.63 | Middle SDI |
| Nicaragua | 0.53 | Low-middle SDI |
| Panama | 0.68 | Middle SDI |
| Venezuela | 0.66 | Middle SDI |
| **Tropical Latin America** | 0.66 | Middle SDI |
| Brazil | 0.66 | Middle SDI |
| Paraguay | 0.62 | Middle SDI |
| **North Africa and Middle East** | 0.64 | Middle SDI |
| Algeria | 0.7 | High-middle SDI |
| Bahrain | 0.71 | High-middle SDI |
| Egypt | 0.6 | Low-middle SDI |
| Iran | 0.7 | High-middle SDI |
| Iraq | 0.58 | Low-middle SDI |
| Jordan | 0.7 | High-middle SDI |
| Kuwait | 0.79 | High-middle SDI |
| Lebanon | 0.73 | High-middle SDI |
| Libya | 0.76 | High-middle SDI |
| Morocco | 0.58 | Low-middle SDI |
| Palestine | 0.54 | Low-middle SDI |
| Oman | 0.74 | High-middle SDI |
| Qatar | 0.77 | High-middle SDI |
| Saudi Arabia | 0.78 | High-middle SDI |
| Syria | 0.61 | Middle SDI |
| Tunisia | 0.68 | Middle SDI |
| Turkey | 0.73 | High-middle SDI |
| United Arab Emirates | 0.79 | High-middle SDI |
| Yemen | 0.43 | Low SDI |
| Afghanistan | 0.29 | Low SDI |
| Sudan | 0.48 | Low-middle SDI |
| **Central Sub-Saharan Africa** | 0.46 | Low-middle SDI |
| Angola | 0.46 | Low-middle SDI |
| Central African Republic | 0.33 | Low SDI |
| Congo | 0.57 | Low-middle SDI |
| Democratic Republic of the Congo | 0.36 | Low SDI |
| Equatorial Guinea | 0.63 | Middle SDI |
| Gabon | 0.65 | Middle SDI |
| **Eastern Sub-Saharan Africa** | 0.39 | Low SDI |
| Burundi | 0.31 | Low SDI |
| Comoros | 0.43 | Low SDI |
| Djibouti | 0.48 | Low-middle SDI |
| Eritrea | 0.41 | Low SDI |
| Ethiopia | 0.33 | Low SDI |
| Kenya | 0.5 | Low-middle SDI |
| Madagascar | 0.33 | Low SDI |
| Malawi | 0.35 | Low SDI |
| Mozambique | 0.34 | Low SDI |
| Rwanda | 0.41 | Low SDI |
| Somalia | 0.23 | Low SDI |
| Tanzania | 0.41 | Low SDI |
| Uganda | 0.39 | Low SDI |
| Zambia | 0.47 | Low-middle SDI |
| South Sudan | 0.27 | Low SDI |
| **Southern Sub-Saharan Africa** | 0.64 | Middle SDI |
| Botswana | 0.66 | Middle SDI |
| Lesotho | 0.49 | Low-middle SDI |
| Namibia | 0.62 | Middle SDI |
| South Africa | 0.68 | Middle SDI |
| Swaziland | 0.58 | Low-middle SDI |
| Zimbabwe | 0.46 | Low-middle SDI |
| **Western Sub-Saharan Africa** | 0.44 | Low SDI |
| Benin | 0.37 | Low SDI |
| Burkina Faso | 0.28 | Low SDI |
| Cameroon | 0.48 | Low-middle SDI |
| Cape Verde | 0.55 | Low-middle SDI |
| Chad | 0.25 | Low SDI |
| Cote dIvoire | 0.41 | Low SDI |
| The Gambia | 0.4 | Low SDI |
| Ghana | 0.54 | Low-middle SDI |
| Guinea | 0.32 | Low SDI |
| Guinea-Bissau | 0.35 | Low SDI |
| Liberia | 0.33 | Low SDI |
| Mali | 0.27 | Low SDI |
| Mauritania | 0.47 | Low-middle SDI |
| Niger | 0.19 | Low SDI |
| Nigeria | 0.49 | Low-middle SDI |
| Sao Tome and Principe | 0.49 | Low-middle SDI |
| Senegal | 0.37 | Low SDI |
| Sierra Leone | 0.36 | Low SDI |
| Togo | 0.41 | Low SDI |

SDI, socio-demographic index.
